# Supplementary material for: Stakeholder Views of the Proposed Introduction of Next Generation Sequencing into the Cystic Fibrosis Screening Protocol in England
Source: Int J Neonatal Screen. 2024 Feb 14;10(1):13. doi: 10.3390/ijns10010013 (PMC10885054; doi:10.3390/ijns10010013)
Supplement: Supplementary file 1 [file IJNS-10-00013-s001.zip › IJNS-2807909-supplementary.pdf]

CF NBS Diagnosis Story

This story contains themes that some people may find challenging or upsetting. These include experiences of receiving a positive result from cystic fibrosis newborn screening, and the reactions of parents to their child's subsequent diagnosis. The characters in the stories are fictional, but the themes, and quotes are directly derived from research. As such, these stories reflect 'real world' attitudes and experiences.

If you feel that reading and/or discussing this story in the context of a focus group would be difficult for you, please contact the researcher, Pru Holder (Pru.Holder@city.ac.uk), and arrangements can be made for you to opt out of this part of the discussion. You may also contact the research team if you wish to discuss your reaction to the story prior to the focus group. At the start of the focus group, all participants will be reminded of the need to treat the views and experiences of others with respect and compassion, even if they are different to their own.

Should you feel distressed before, during or after the focus group, or would like further information or support regarding cystic fibrosis newborn screening and its impacts, please contact the Cystic Fibrosis Trust (0300 373 1000 or helpline@cysticfibrosis.org.uk) or your clinical team, where appropriate.

CF Diagnosis Story

Rhiannon (40) and Michael (48) already had a daughter (Evelyn, age 4), when their second child, a son they named Jacob, was born in 2018. The family were living in a rural part of South West England at the time, and Jacob was born in a small hospital, local to their home. Jacob's birth was straightforward, and after an overnight stay in hospital, Rhiannon and Jacob were discharged home. Rhiannon described the following days as '*busy*' and '*exhausting*', but nevertheless '*wonderful*' as they adjusted to becoming a family of four. Jacob lost some weight during these early days which Rhiannon mentioned to the visiting midwife, but she was given reassurance that this was common after birth, and that Jacob would regain the lost weight as breast feeding became better established. When Jacob was 17 days old, however, Rhiannon described receiving an unexpected phone call from a CF nurse specialist;

It was a complete bolt from the blue- completely. You know, the last thing you'd expect because…I'd had such a straightforward pregnancy, the birth was…fine …I actually missed the calls as they'd been ringing the landline and I'd got all my 'congratulations!' cards all around the answering machine, I never saw it flashing. Been too busy, enjoying my new baby, you know? People were visiting. By the

time they got hold of me they were already on their way round to the house, and this CF nurse turned up, handed me a leaflet on CF on the doorstep and said, 'I need to give you this…can I come in?'

(Rhiannon)

Rhiannon described being on her own with Evelyn and Jacob during this visit, as Michael had returned to work following his paternity leave. The CF nurse explained that the screening result had come back positive for CF, and that Jacob needed more tests. Rhiannon remembered the heel prick test being done, but hadn't thought much about it. She wasn't aware what was being testing for, other than them being '*fairly obscure genetic conditions*';

I just thought "oh, well, it can't be that [CF] because surely, you know, we'd have a family history or he'd sound really wheezy or something."

(Rhiannon)

The CF nurse left Rhiannon with some information on cystic fibrosis, and asked her to bring Jacob to a CF centre based in a large city hospital that was 45 miles from their home the next morning to meet with a paediatric consultant. Before she left, she asked that Rhiannon bring Michael with her to the appointment, which Rhiannon found alarming;

I knew when she said 'bring your husband' that it wasn't good. My mind was just racing, and I rang Michael…and we were scrambling to read as much about CF as we could that night, even though they had said to us, 'don't google it, just come to see us'…. But of course you do anyway. You're in panic mode. Maybe it was good we didn't have long to do it, we were at the hospital the next day having the sweat test. Up until that point, we'd still got a bit of hope that they'd got it wrong, but then they said, 'no, this is what he's got'. And they admitted him to hospital there and then to start him on creon and antibiotics. Our world just collapsed that day, and I just couldn't take it all in. I was his mum- how could I have missed that something was so terribly wrong? It wasn't even that I was new at this, he was my second. And so we went from having a healthy baby to a very sick one in 48 hours.

(Rhiannon)

Michael described his reaction to Jacob's diagnosis and immediate admission for treatment as one of grief, that impacted both the immediate and extended family;

….it was just grief, really. A sort of terrible feeling of, you know, "why him, and not us?". And guilt, somewhat. And just being really scared. I mean, I had known people with CF, one who passed away and another who's doing a lot better, and he's still alive, but it was, it was just a really scary time. We gave it 24 hours before we told anyone else… the family really struggled. Because it is genetic, they were all wondering if it was them, if they'd caused their children or their grandchildren harm, and that's quite a lot to bear thinking "It's down to you- you're the one that's caused this." Not just having a child sick in hospital, it's also "this is something that you've passed down." So there was a lot of emotion going around. And having that extra 24 hours meant that when we finally did tell the parents and they all rushed to the hospital, we could cope with their questions because they were asking all the similar ones that had already asked. *(Michael)*

Along with reactions of grief, shock and guilt, Michael also reflected on how their attention quickly turned to their future life plans;

….and would you believe this all happened when we were moving house…the next day [after diagnosis] we were due to exchange. And so then it was going through our minds, "Are we doing the

right thing? Can we afford to move? What if Rhiannon can't go back to work now? Can we afford the mortgage?" So there were a million and one things going through our minds at that time.

(Michael)

Despite the initial shock of Jacob's diagnosis, both Rhiannon and Michael emphasised how important they considered newborn screening for cystic fibrosis to be. For Rhiannon, this was because Jacob was immediately started on treatment;

I have to say, he was a different baby once he was on the creon. I think he gained a pound in the first week. So really up until that point, those first three of four weeks, he had not been digesting the milk at all …but as soon as he was on creon, he started gaining weight really fast. He went from being a miserable baby to a very satisfied, happy one. So things like that, getting started on treatments as early as you can, can stop them from getting very poorly. I think it's important especially now with all the new treatments coming out- the sooner you can get them started on them the better, I think.

(Rhiannon)

Michael also mentioned the benefits of early diagnosis through newborn screening in terms of reproductive planning;

I would say we had the reaction of, "that's it, we're not gonna have any more children." We talked it through, and we were made up on that because the children that have more than one CF kid in the family, we read that they are generally in worse health, because they pass the different bacteria and whatnot on to each other. Particularly pseudomonas- if you've got it in one, you're gonna get it in the other. So we were glad that we found out early…because, with our age, we would have gone for a third [child] immediately, and this changed things- just in time really. We didn't want to be in the position of making Jacob's health worse by having another [child with CF], and two with CF would have been a lot to cope with.

(Michael)

CF Clinical Diagnosis Story

This story contains themes that some people may find challenging or upsetting. These include experiences of receiving a delayed diagnosis of cystic fibrosis and of having a child with the condition, told from a parent's perspective. The characters in the stories are fictional, but the themes, and quotes are directly derived from research. As such, these stories reflect 'real world' attitudes and experiences.

If you feel that reading and/or discussing this story in the context of a focus group would be difficult for you, please contact the researcher, Pru Holder (Pru.Holder@city.ac.uk), and arrangements can be made for you to opt out of this part of the discussion. You may also contact the research team if you wish to discuss your reaction to the story prior to the focus group. At the start of the focus group, all participants will be reminded of the need to treat

the views and experiences of others with respect and compassion, even if they are different to their own.

Should you feel distressed before, during or after the focus group, or would like further information or support regarding cystic fibrosis newborn screening and its impacts, please contact the Cystic Fibrosis Trust (0300 373 1000 or helpline@cysticfibrosis.org.uk) or your clinical team, where appropriate.

CF Clinical Diagnosis story

Vicki (now 52) is mother to Annabelle, who was born in a large hospital in a city in the North East of England in December 2000, a time when newborn screening for cystic fibrosis had not yet been introduced. Vicki and her partner Ben (now 55), Annabelle's father, separated during Vicki's pregnancy but remained on good terms and co-parented their daughter. Annabelle's birth was uncomplicated, with Vicki stating that they both '*bounced back really quickly*'. However, within a week of returning home with her new baby, Vicki began to notice what she describes as *'a few issues'* with Annabelle, that she found hard to pinpoint at first;

It was difficult to say what was wrong at first because the symptoms that she had then were a bit vague- they were things that lots of babies go through and could have been down to anything. Like she seemed to be in awful pain after feeding and she'd cry and howl and pull her knees up like it hurt, and the health visitor just said 'It's just colic-you need to ride it out' which was… helpful [laughs]. But Annabelle also wasn't putting much weight on as well- she had gained some, but it was hardly anything- so I took her to the GP and he just said 'this is normal for this age, don't worry about it, you know, she'll catch up'.

(Vicki)

Annabelle continued to have difficulties with feeding and weight gain, and at three months of age, she became unwell with a chest infection that resulted in her being admitted to hospital for IV antibiotics. Vicki remembered their experience of the hospitalisation well;

She'd got this chest infection, and I took her to straight to A & E as I could tell she was struggling, and they admitted her. But the problem was, this was on a Good Friday…it was over the Easter weekend, and I don't think...you know, the main doctors, the specialists weren't in, they were all on holiday and they just had skeletal staff to cover it… and no one could really answer the questions I had about why she was still colic-y, why she was so skinny, they just gave her antibiotics and oxygen and sent us home on the Easter Monday. They fobbed us off basically.

(Vicki)

This first chest infection ended up being a pivotal moment in Annabelle's life, as it triggered what Vicki described as a *'rapid and immediate decline'* in her health;

From that point on, the chest infections were pretty much constant, and I was never sure whether it was a new one each time, or it was that first one still that she hadn't managed to clear, but I kept going back to the doctors- it was at least once a week by then. The GP didn't seem that worried about her, and on about the fifth or sixth appointment he [GP] looked me in the eye and said 'you

need to stop doing this, you're becoming neurotic about it' you know, 'we can't keep giving her endless antibiotics'. He kind of insinuated that it was because I kept giving antibiotics that I wasn't letting her immune system develop. So he put it back on me, which was incredibly damaging. And it didn't help that Ben [Annabelle's father] was only seeing her every other weekend, and of course he tended to see her when she was a bit better, and so he didn't believe how poorly she'd been in between.

(Vicki)

Vicki described the emotional distress she experienced at being dismissed by both the GP and her ex-partner and how *'alone'* she felt in caring for Annabelle. From that point on, she became *'embarrassed'* about asking for help (from both health professionals and family), and ultimately became depressed.

Looking back, it was depression really, probably post-natal depression mixed in with this horrible feeling that everyone was either ignoring me, or blaming me for doing the wrong thing by Annabelle. I really took it all to heart, and I even began to wonder myself if I had Munchausen's by-proxy or something, I felt like I was going mad!

(Vicki)

By six months of age, Annabelle's weight had dropped to below the 5th centile, and Vicki described her as having *'awful greasy nappies'* that weren't improving. However, she still felt reluctant to seek help given the GP's comments about her. She even feared that Annabelle might be *'taken away'* by social services if she kept going back. In the end, it took a family member coming to visit to empower Vicki to take control of her daughter's health;

…It took my sister coming over one day- and she lives 150 odd miles away, so she hadn't seen Annabelle often- it took her coming over and saying 'right enough of this, we're going straight up to the hospital'. I think the shock of seeing how poorly Annabelle was from when she'd last seen her, just triggered this 'right we need to sort this out immediately' attitude. I said to her, 'look, they won't do anything, you know, I've asked…', but because she was so adamant, it gave me the courage. When we got there, we saw this new paediatrician bloke and he was so different, he took one look at Annabelle and admitted her on the spot. He said she was too thin, her chest wasn't right, he put her on an IV and she started getting the enzymes and antibiotics, and that really made the world of difference. It was such a relief, because we had both been in such a bad place mentally and physically, and to have someone say 'yes', you know, 'I see what you see, you're not crazy'. And so that was how it got diagnosed really, once she was improving, they did the sweat test and that came back positive, and I think she had just turned eight months by then.

Following a confirmed diagnosis of cystic fibrosis, Annabelle began to receive specialist care which made a significant difference to her health. Vicki reported feeling like her sister and the paediatrician had been their *'saviours'*, getting them on the *'right path'* of treatment, and validating Vicki's concerns. However, she continued to have concerns that Annabelle's difficult, and long, route to diagnosis may have longer term impacts for her health. When considering the cystic fibrosis newborn heel prick test, that was introduced seven years after Annabelle was born, Vicki reflected;

I do think it would have saved me and her a lot of pain and suffering if it had been picked up at birth. Those first eight months would have been so different if we'd been told straight away and could have got her onto treatment. She'd got to the point of being so poorly before anyone took it seriously, and yeah, it did cause me to have a bit of a breakdown actually. I felt very alone and like I couldn't look

after my daughter properly and no one believed me- including Annabelle's father. And that was all unnecessary really if it could have been picked up by a simple blood test.

(Vicki)

When considering if there are any 'down sides' to receiving an early diagnosis, Vicki commented;

I guess it [diagnosis] would have been more of a shock back then [after birth] than it was in the end [at eight months]. Because she was very unwell by the time we found out, all I felt was just… relief at first- you know, as horrible as CF is, at least now we can do something about it. I'm not sure what my reaction would have been if she'd been perfectly ok, and then they'd told me 'well sorry, but she's got CF'. It probably would have been more scary. But I think if you've got a solid diagnosis at the outset then people support you. You know, my family would have all been there and rallied round, and I think Ben would have acted differently. Having that support system in place is so important, and you need that diagnosis to make everything else fall into place, then you can cope a lot better. Better than I did anyway!

(Vicki)

CFSPID story

This story contains themes that some people may find challenging or upsetting. These include experiences of receiving a positive result from cystic fibrosis newborn screening and the reactions of parents to their child's CFSPID designation. The characters in the stories are fictional, but the themes, and quotes are directly derived from research. As such, these stories reflect 'real world' attitudes and experiences.

If you feel that reading and/or discussing this story in the context of a focus group would be difficult for you, please contact the researcher, Pru Holder (Pru.Holder@city.ac.uk), and arrangements can be made for you to opt out of this part of the discussion. You may also contact the research team if you wish to discuss your reaction to the story prior to the focus group. At the start of the focus group, all participants will be reminded of the need to treat the views and experiences of others with respect and compassion, even if they are different to their own.

Should you feel distressed before, during or after the focus group, or would like further information or support regarding cystic fibrosis newborn screening and its impacts, please contact the Cystic Fibrosis Trust (0300 373 1000 or helpline@cysticfibrosis.org.uk) or your clinical team, where appropriate.

CFSPID Story

Rory (37) and Laila (31) are first time parents to baby Grace, who was born following a straightforward delivery at a large city hospital in the North West of England. Once the couple returned home with baby Grace, they were keen to introduce her to their friends and family. The midwife visited them at home daily for the first five days, but Rory had no memory of the heel prick test ever being done during those visits, which only heightened his level of shock when Laila received a phone call saying something had been found in the results related to cystic fibrosis:

It was...really odd really, how they did it. They just sort of rang Laila up…when, you know, she'd not long given birth and we were...just getting to grips with being parents, first time around...someone rang her up and said 'something's come up on the bloodspot', and 'can you come in?'. And I didn't [laughs], perhaps I wasn't paying attention, but I didn't realise she'd [Grace] even had the thing done….so this really was a shock for me!

(Rory)

Laila also reacted with shock, and immediately rushed to investigate what cystic fibrosis was. They were seen at the hospital the very same day to have further testing. However, Laila described the whole process, and the urgency around it as *'traumatic'*;

I think it's every parent's worst nightmare really, being told 'we've found something that needs investigating'. And we were lucky…they saw us the same day, but even as we were going to [hospital] I was googling CF, and by the time we'd arrived at the doors I'd convinced myself that she was dying and I was just a bit of a mess.

(Laila)

When the results of these further tests came through, Rory and Laila were informed that Grace had the CFSPID designation. For the couple, this designation challenged their ideas about health, illness and the role of medicine. Rory explained;

It [CFSPID]might be something…or it might be something of nothing. They just can't tell you. …I thought that medicine was more black and white than that- you either have something, or you don't. Even if it's mild, it's still one thing or another. But apparently that's not true!

(Rory)

The uncertainty of the designation made it hard for the couple to adjust to, and accept, the information about Grace's health;

I mean obviously although I'm glad she doesn't have full CF, but we have to live with the possibility of it every day. You know, how I parent her…I will always have one eye in that direction… 'if this [CF] happens, will she be able to cope with x,y,z?'. We factor it in to the decisions we make as a family. You can't 100% worry about it, or 100% ignore it, it's just the perpetual in-between.

(Laila)

Following the result, Grace began to be invited for a health review once a year at the paediatric CF centre based in the city in which she was born. Whilst Laila and Rory found the team there to be *'great'* (Rory) and the annual check-ups *'reassuring'* (Laila), they could also cause the couple some worry- particularly when they knew an appointment was coming up. Laila and Rory also found it hard to explain Grace's designation to others- including their own family- who struggled to understand why Grace didn't have CF, yet was being monitored by a CF team.

I find it really hard to explain to people, so we often just call it a rare type of CF, which I suppose it is...but like…my parents, I don't think even they fully understand what it is. They don't know whether they should be keeping her in on cold days in case she catches a cold that might set it off, or if they should let her get on with it. For me, I find I can forget about it most days, and we don't tend to…stop her doing the things she wants to do because of it. But when it comes to a check-up, or she gets chesty, you're worrying about it more ….in the weeks running up to an appointment [at CF centre] I tune in to it, start monitoring her for any signs, start seeing CF in everything. You can get a bit paranoid about it, so yeah that part's difficult.

(Laila)

As well as difficulties with explaining the designation to friends and family, Rory felt that the uncertainty around CFSPID meant that they didn't *'fit in'* anywhere, including within social circles familiar with CF;

"We don't really fit into either camp- either the CF world, or the healthy kid world. Other parents don't get it. We don't belong fully in either place."

(Rory)

However, as time went on, and Grace remained well, the couple's sense of CF risk began to alter. They reflected on how they had felt most concerned about CFSPID when Grace was small and *'vulnerable'*, but now describe her as *'fairly robust'*;

…we're both a bit less worried than you know we were a few years ago, now she's a bit older and she's doing absolutely fine- I'd say she's thriving!

(Rory)

When considering whether or not they are glad that they were told about Grace's CFSPID designation following newborn screening, Rory and Laila had differing views, Rory stated;

Laila likes to prepare, but I think…I like to not know things sometimes. If you know too much, you worry, you see.

(Rory)

Laila on the other hand, saw benefits to receiving the information:

I'm one of those people who likes to have full facts…I feel safer having all the information…….because knowledge is power, and now we can put things in place for Grace, now that we know the full range of possibilities for her [future] health.

(Laila)

CF Carrier Story

This story contains themes that some people may find challenging or upsetting. These include experiences of receiving a positive result from cystic fibrosis newborn screening, and the reactions of parents to their child's carrier status. The characters in the stories are fictional, but the themes, and quotes are directly derived from research. As such, these stories reflect 'real world' attitudes and experiences.

If you feel that reading and/or discussing this story in the context of a focus group would be difficult for you, please contact the researcher, Pru Holder (Pru.Holder@city.ac.uk), and arrangements can be made for you to opt out of this part of the discussion. You may also contact the research team if you wish to discuss your reaction to the story prior to the focus group. At the start of the focus group, all participants will be reminded of the need to treat the views and experiences of others with respect and compassion, even if they are different to their own.

Should you feel distressed before, during or after the focus group, or would like further information or support regarding cystic fibrosis newborn screening and its impacts, please contact the Cystic Fibrosis Trust (0300 373 1000 or helpline@cysticfibrosis.org.uk) or your clinical team, where appropriate.

CF Carrier Story

Hannah (28) and Arun (31) had their first child, Noah, in 2017 whilst living in a town on the South East coast of England. Noah was born at 39 weeks via caesarean section, and Hannah needed a blood transfusion after the delivery, but three days later they were discharged home from the hospital. Hannah vividly remembered Noah's heel prick test being done, but didn't remember consenting to it;

I remember thinking it was a horrible test, because it's not a 'prick'- they take a little blade to your baby's foot! I remember feeling really upset, and I didn't want it done, but he is my first, and I didn't think I had much choice about it. I wanted to do the best thing for him. The midwife came out to take my stitches out [from the caesarean] and said 'oh, and while I'm here, I need to do the heel prick'. They made it sound like it was mandatory, so you just go along with it…you don't say yes, they just do it.

(Hannah)

Despite finding the heel prick test an unpleasant experience, Hannah said she soon *'forgot about it'*, until she received a phone call two weeks later saying that there was a *'problem with the test'*;

I was at a baby group, and when I looked at my phone I'd got three missed calls. They'd left a message saying someone needed to speak to me about Noah's result and I felt sick to my stomach. When I spoke to them, they said they'd already sent someone round to the house to do another heel prick but we'd been out, so I had to rearrange that. They didn't say it could be CF at that point, they just said there was a 'problem with the test', so I thought it was a technical thing. It was only when they came and took the blood the second time that they mentioned CF, and I lost the plot. I was on my own because I thought they were just re-doing the test. All I knew of CF was that it makes it really hard for people to breathe, but the health visitor didn't seem concerned at all that she'd dropped this bombshell.

(Hannah)

Hannah described a frantic search for information about cystic fibrosis as she and Arun waited for the results of the second heel prick test. This included speaking to family members, which uncovered a possible family history of cystic fibrosis that Hannah hadn't previously considered as relevant;

When I spoke to my mum, it turns out a great uncle of mine had died when he was 2 or 3 from some respiratory illness, and she'd heard someone say once that it could have been cystic fibrosis but they wouldn't have known that at the time… they put it down to Tuberculosis. But the story has always been told in my family as it was so tragic how young he died, I just never thought about it all that much before. So it was super stressful. With the family history, or the potential family history, the wait for the results was agonising.

(Hannah)

Hannah and Arun were phoned the following week by a CF nurse to receive the results of the second test. The nurse explained that Noah was found to be a 'probable carrier' of cystic fibrosis, and that arrangements could be made for the couple to see a genetic counsellor if they wanted. Hannah and Arun accepted the offer as they thought it would be a good opportunity to get a more in depth understanding of what probably carrier status is, and what it might mean for Noah's life. Hannah described her reactions to Noah's result, and the genetic counselling session, in the following way;

The first feeling is a flood of relief because you know, he's not going to die of this. This isn't cystic fibrosis, which is what we thought they were testing for, you know? It's not in the forefront of our minds now, but we do know now that he has this thing, and that when he comes to have his own kids, that's when it comes out. If it's as common as they say [CF carrier status], it is possible he will meet another [carrier] and well….he's going to have be more careful than most about any unplanned pregnancies! He might need to tell any future partners because there's going to need to be blood tests done before having kids, or he might choose not to tell anyone at all! But it would probably be sensible, and I'd encourage that.

(Hannah)

Arun similarly responded with overwhelming relief, but once the news had *'sunk in'*, he began to wonder about how cystic fibrosis carrier status might affect Noah's life,

I'll be honest with you, I'd never heard of a carrier before…. I was genuinely worried that he might feel like he was different because of this. And we've had a think about how, and when, might be the

best time to tell him that won't make him feel that way. Because it's not really a diagnosis is it? It's just information, and it won't be relevant to him for a very very long time, I just don't want him to feel that something is wrong with him.

(Arun)

Hannah and Arun both felt that it was important to communicate Noah's carrier status result to their extended families. Hannah's siblings both opted to be tested, and one was found to be a carrier. Hannah reflected on this;

Arun and I are both the youngest in our sibling groups, so most of them had already had their children by the time we found out, but my older brother…he wanted to know for his daughters and his came back positive, whereas my younger sister was negative. It just means that my nieces have now got that information, you know, if they want to be tested in the future, they have that option. There are things available now that weren't available to previous generations, so they are fortunate really to be able to find out from the get go.

(Hannah)

Arun reflected on his family's response to Noah's carrier status in the following way;

It did mean….and I'm from a big family, our family is….huge [laughs] so it did mean I had to tell them all-which took ages! But mostly they didn't take it too seriously, I think because it's not a diagnosis …if they did take tests, they didn't tell me about it anyway.

(Arun)

Despite her difficult experience with the repeated heel prick test, Hannah thought that overall it was useful for her to know about Noah's carrier status;

Yeah I do think you should know about this sort of thing- even if the way of finding out was pretty…rubbish actually. You know, it made us think about the family history differently, and it's empowering for the next generation as now we know it's there, they can do something about it if they want to.

(Hannah)

Arun on the other hand, had more ambivalent feelings about the value of being told about carrier status early in life.

Yes…I mean yes it's good to know, but it felt like the wrong time ….the impact it had on our mental health, on Hannah's mental state during those weeks was quite bad…you know? We thought Noah could be dying and it took the….shine off having a new baby. It came out ok in the end, but perhaps it was information that could have waited until later, when Noah actually needs it himself.

(Arun)
